# The Diagnostic and Prognostic Value of Plasma Galectin 3 in HFrEF Related to the Etiology of Heart Failure

**DOI:** 10.3389/fcvm.2021.748875

**Published:** 2021-12-22

**Authors:** Qun Lu, Ruo-Chen Zhang, Shu-Ping Chen, Tao Li, Ya Wang, Yan-Bo Xue, Jing Liu, Xiu Han, Yi-Dan Su, Ling Bai, Xiao-Jun Du, Ai-Qun Ma

**Affiliations:** ^1^Department of Cardiovascular Medicine, First Affiliated Hospital, School of Medicine of Xi'an Jiaotong University, Xi'an, China; ^2^Department of Cardiovascular Medicine, Xi'an Central Hospital, Xi'an, China; ^3^Experimental Cardiology Lab, Baker Heart and Diabetes Institute, Melbourne, VIC, Australia; ^4^Department of Physiology and Pathophysiology, Medical College of Xi'an Jiaotong University, Xi'an, China

**Keywords:** heart failure, galectin-3, prognosis, risk factor, diagnostic, etiology

## Abstract

**Aim:** The aim of present study is to evaluate the diagnostic and prognostic value of plasma galectin 3 (Gal-3) for HF originating from different causes.

**Methods:** We investigated the plasma levels and expression of Gal-3 in cardiac tissues in two transgenic (TG) strains of mice with cardiomyocyte-restricted overexpression of either β2- adrenergic receptor (β2- AR TG) or Mammalian sterile 20-like kinase 1 (Mst1-TG) in the present study. Additionally, 166 patients suffering from heart failure with reduced ejection fraction (HFrEF) in two hospitals within the Shaanxi province were examined in this study. All these patients were treated according to the Chinese HF guidelines of 2014; subsequently, they were followed up for 50 months, and we analyzed the prediction value of baseline Gal-3 to endpoints in these patients.

**Results:** Gal-3 was localized in the cytoplasm and nucleus of cardiomyocytes, often formed aggregates in Mst1-TG mice. Extracellular Gal-3 staining was uncommon in Mst1-TG hearts. However, in β2-AR TG mice, although Gal-3 was also expressed in myocardial cells, it was more highly expressed in interstitial cells (e.g., fibroblasts and macrophages). Plasma Gal-3 was comparable between nTG and Mst1-TG mice. However, plasma Gal-3 was higher in β2-AR TG mice than in nTG mice. In the cohort of HFrEF patients, the median plasma Gal-3 concentration was 158.42 pg/mL. All participants were divided into two groups according to Gal-3 levels. Patients with Gal-3 concentrations above the median were older, and had lower plasma hemoglobin, but higher plasma creatinine, tissue inhibitor of metalloproteinases 1 (TIMP-1), left ventricular end systolic diameter (LVESD), left ventricular end-systolic volumes (LVESV) and end-diastolic, as well as left ventricular end-diastolic volumes (LVEDV). Spearman correlation analysis revealed that Gal-3 was positively correlated with TIMP-1 (*r* = 0.396, *P* < 0.001), LVESV (*r* = 0.181, *P* = 0.020) and LVEDV (*r* = 0.190, *P* = 0.015). The 50-month clinical follow-up revealed 43 deaths, 97 unplanned re-hospitalizations, and 111 composite endpoint events. Cox analysis demonstrated that although Gal-3 did not provide any prognostic value in either total-HF subjects or coronary-heart-disease (CHD) patients, it did provide prognostic value in non-CHD patients.

**Conclusion:** Although plasma Gal-3 is associated with TIMP-1 and echocardiographic parameters, the diagnostic and prognostic value of Gal-3 in HFrEF is determined by the etiology of HF.

## Highlight

The diagnosis and prognostic value of Galectin-3 in HFrEF.

## Introduction

Heart failure (HF) is a disease of high morbidity and mortality regardless of therapies (1, 2). Hence, there is an increasing demand for early diagnosis, better prognostic evaluation and management of HF. Thus, as indicators of pathological processes and responses to therapeutic interventions, circulating blood biomarkers have been increasingly studied due to their non-invasive determinations that tend to be sufficiently sensitive and accurate. Due to different etiologies and underlying pathophysiological processes, HF was a heterogeneous disease. Although there are many different available biomarkers [e.g., NT-proBNP (3), GDF-15 (4)], the diagnosis and prognostic values of these biomarkers were discordant.

Galectin 3 (Gal-3) is a soluble β-galactoside-binding protein, expressed in epithelial and inflammatory cells in several organs, located both intracellularly and extracellularly (5, 6), involved in cellular functions related to cell adhesion (7, 8), proliferation (9) and differentiation (10–12), the Gal-3 is considered as a biomarker of cardiac fibrosis and remodeling (5, 13, 14). In the myocardium, Gal-3 is primarily expressed in fibroblasts and macrophages that play an important role in the formation of myocardial fibrosis through the activation of fibroblasts (15), and have been linked to fibrosis in a spectrum of medical conditions, including HF. Although many previous studies have demonstrated elevated plasma concentrations of Gal-3 in both acute and chronic HF, the prognostic value of Gal-3 in predicting re-hospitalization and mortality has not yet been determined. Zhao et al. (16) and we (17) found that plasma and cardiac levels of Gal-3 were different across distinct HF caused by different etiologies in experimental animals. Therefore, it seems reasonable to hypothesize that the diagnostic and prognostic value of plasma Gal-3 is related to and affected by the etiology. The researchers of this study, therefore, evaluated the diagnosis and prognostic value of plasma Gal-3 within HF patients caused by different causes.

## Methods

This study was conducted in mouse models and HF patients.

### Animals

Two transgenic (TG) strains of mice with cardiomyocyte-restricted overexpression of either β2- adrenergic receptor (β2- AR TG) or Mammalian sterile 20-like kinase 1 (Mst1-TG) were used in the present study. Our previous works have characterized cardiomyopathic phenotypes of both models (16, 18–20). These two strains of mice were both from the C57Bl/6 genetic background and female mice were excluded. Age-matched non-transgenic (nTG) littermates were used as controls. Mice were housed in standard conditions with food and water provided *ad libitum*. All experimental procedures were approved by a local animal Ethics Committee in compliance with both the Australian Code for the Care and Use of Animals for Scientific Purposes (8th edition) and the ARRIVE guidelines.

### Subjects

This protocol was approved by the Ethics Committee of the First Affiliated Hospital of Xi'an Jiaotong University (Shaanxi 710061, China, approval number: 2013-133) and was in accordance with the Helsinki Declaration's guidelines. Informed consent was obtained from all participants. The cohort study consisted of chronic HF patients, aging between 18 and 80 years, who were diagnosed with heart failure with reduced ejection fraction (HFrEF) in the Department of Cardiovascular Medicine at Xunyi Hospital and Jingyang Hospital from May 2014 to May 2015 by the physicians who were blinded to the study. According to China HF guidelines of 2014, diagnosis of HFrEF was based on: ① typical symptoms and signs, ② clinical history, ③ NT-proBNP ≥125 pg/mL, ④ left ventricual ejection fractions <40%. Patients were excluded from the present study if they had acute HF, active neoplasia, acute myocardial infarction, acute or chronic liver disease (alanine aminotransferase level >5 times the upper normal limit), acute stroke, serious kidney disease, chronic consumption disease, thyroid dysfunction, fibrotic pathologies (e.g., pulmonary fibrosis, collagenases), valvular heart disease and/or cancer. A total of 166 HFrHF patients were recruited to satisfy these exclusion criteria. All participants were divided into two groups according to the media level of Gal-3. Then, based on their clinical features, as well as their past medical history and/or the result of coronary angiography, patients from each group were further divided into two subgroups, namely, the group of those with coronary-heart-disease (CHD) group and the group of those without CHD group. Patients were then followed up for a period of 50 months and were evaluated for the development of major adverse cardiovascular events (MACEs).

### Plasma and Histological Gal-3 Analysis in Mice

Blood was collected in heparin-containing vials when mice were killed, centrifuged at 4°C (3,000 rpm in 20 min), and stored at −80°C. Plasma Gal-3 levels were detected by a mice Galectin-3 Quantikine ELISA Kit (R&D Systems Inc., Minneapolis, MN, USA) in twin duplicates wells, following protocols provided by the manufacturer. The detection range of the plasma Gal-3 was 8.19–2,000 pg/mL, and the limit of detection was 6 pg/mL.

Paraffin-embedded LV sections (6 μm) were prepared and used for Gal-3 immunofluorescent staining within mice. For Gal-3 immunofluorescent staining, after dewaxed, heat-induced antigen retrieval and permeabilization were carried out (with 10 mM of Na-citrate buffer containing 0.05% Tween 20; pH 6.0; 95°C for 25 min) followed by blocking with DAKO Protein Block (X0909, Agilent, 1 h at room temperature). Sections were incubated with primary goat anti-mouse Gal-3 (1:100, AF1197, R&D Systems) overnight at 4°C, then they were incubated with the secondary antibody, Alexa Fluor 594 donkey anti-goat IgG (1:200, A11058, Invitrogen by Thermo Fisher Scientific). The cardiomyocyte boundary was revealed by wheat-germ-agglutinin FITC staining (1:80, FL-1021, Vector Labs, 1 h at room temperature). Images were acquired with an Olympus BX61 fluorescent microscope.

### Clinical Measurements

Investigators and a trained interviewer collected all of the clinical data. At baseline, all medical visits to the patients were conducted and patients' information was collected, including demographic data, past medical history, history of cardiovascular diseases, the Minnesota Living with Heart Failure Questionnaire (MLHFQ), New York Heart Association (NYHA) functional class, smoking behavior, and alcohol abuse. Smoking was defined as smoking cigarettes within one month of the indexed hospital admission. Hypertension was defined as a cuff blood pressure ≥140/90 mmHg and/or the current use of antihypertensive medications. Subjects were also questioned about their past histories of diabetes mellitus and their current use of anti-diabetic drugs. Diagnosis of diabetes mellitus (DM) was confirmed if plasma fasting glucose was ≥7.0 mM (or if the 2-h postprandial glucose was >11.1 mM) or if there was current use of anti-diabetic medication. Anthropometric measurements, such as body weight (kg) and height (m), were taken during the first visit. Body mass index (BMI) was calculated as weight divided by height squared.

### Analysis of Patients' Blood Parameters

For measurements of hemoglobin (HB), creatinine (CR), urea nitrogen (BUN), alanine aminotransferase (ALT), and aspartate aminotransferase (AST), blood was collected from each patient after gaining the consent. These blood parameters were measured at the Central Clinical Laboratory of the First Affiliated Hospital of Xi'an Jiaotong University. HB was measured with automated cell counters via standard techniques by HST201 (Sysmex, Japan). CR, BUN, ALT and AST were examined by Beckman automatic biochemical analyzer AU5431 (America). After overnight fasting, between 6–7 a.m., blood from the median cubital vein was drawn into ethylenediaminetetraacetic acid (EDTA)-containing tubes, centrifuged at 4°C (3,000 rpm in 10 min) within 2 h after collection and stored at −80°C for later analyses (i.e., to detect Gal-3 and TIMP-1 levels).

Plasma Gal-3 levels and TIMP-1 were detected by a Human Gal-3 Quantikine ELISA Kit (R&D Systems Inc., Minneapolis, MN, USA) and a human TIMP-1 Quantikine ELISA Kit (Abbkine, Inc., China) in duplicates wells following protocols provided by the manufacturer, respectively. The mean plasma Gal-3 and TIMP-1 were calculated as the final level. The detection range of the plasma Gal-3 was 0–4,000 pg/mL. The calibration range of serum TIMP-1 was 31.25–2,000 ng/mL, and the limit of detection was 16 ng/mL.

### Echocardiographs and Electrocardiograms

At baseline, echocardiographs were performed with a Phillips iE33 system by a single trained operator blinded to the Gal-3 plasma concentration of each subject. All Echocardiographs data were analyzed by a single operator to limit inter-observer variability. The following standard parameters were collected: left ventricular end-systolic and end-diastolic volumes (LVESV and LVEDV), left ventricular end-systolic and end-diastolic dimensions (LVESD and LVEDD), and left ventricular fraction shortness (LVFS). The left ventricular ejection fraction (LVEF) was calculated by the Simpson biplane model.

The 12-leads electrocardiographic were made up of three standard limb leads (I, II, and III), augmented limb leads (aVR, aVL and aVF), and six precordial leads (V1, V2, V3, V4, V5, and V6). The QT interval was best measured between the beginning of the Q wave until the end of the T wave in lead II.

### Treatments and Evaluations of Patient Outcomes

All HF patients were actively followed up at times of 1, 3, 6, and 50 months after the initial treatments. The complete follow-up information was gained for all 166 patients (100%). Information was obtained by face-to-face interviews or telephone conversations. Information regarding secondary cardiovascular events and treatments was collected, since the start of the treatment in the present study. Cardiovascular events were defined as MACEs as either the main cause of death, re-hospitalization because of HF, or composite endpoint events. All patients received β-blockers, as well as angiotensin-converting enzyme inhibitors (ACEI) or angiotensin receptor blockers (ARB), according to the China HF guidelines of 2014, unless there were contraindications to these drugs. Mineralocorticoid-receptor antagonists (MRA), diuretics, and digoxin were prescribed to patients who had corresponding indications according to the China HF guidelines of 2014.

### Statistical Analysis

Analyses were performed using SPSS version 13.0. Normally distributed values were presented as mean ± standard deviation (SD), and differences between groups were determined using Student's *t-*tests. Variables with a skewed normal distribution were presented as medians (inter-quartile range), and between-group differences for these variables were determined using Rank-Sum tests. Categorical variables were presented as percentages, and differences between groups were tested using Chi-squared tests. Owing to the skewed distribution of Gal-3, Spearman correlation analysis was used to analyze the relationship between plasma Gal-3 levels and clinical characteristics. MACE-rate estimates were generated via the Kaplan-Meier method. Cox regression modeling was used to assess the relative importance of baseline risk factors to the resulting endpoints. Hazard ratios (HR) with 95% confidence interval (CI), were presented to show the risk of an event when a given factor was present. Significance was defined at the 5% level using a two-tailed statistical test.

## Results

### Myocardial and Serum Gal-3 Expression Levels in Cardiomyopathic Mice

**Figure 1:**
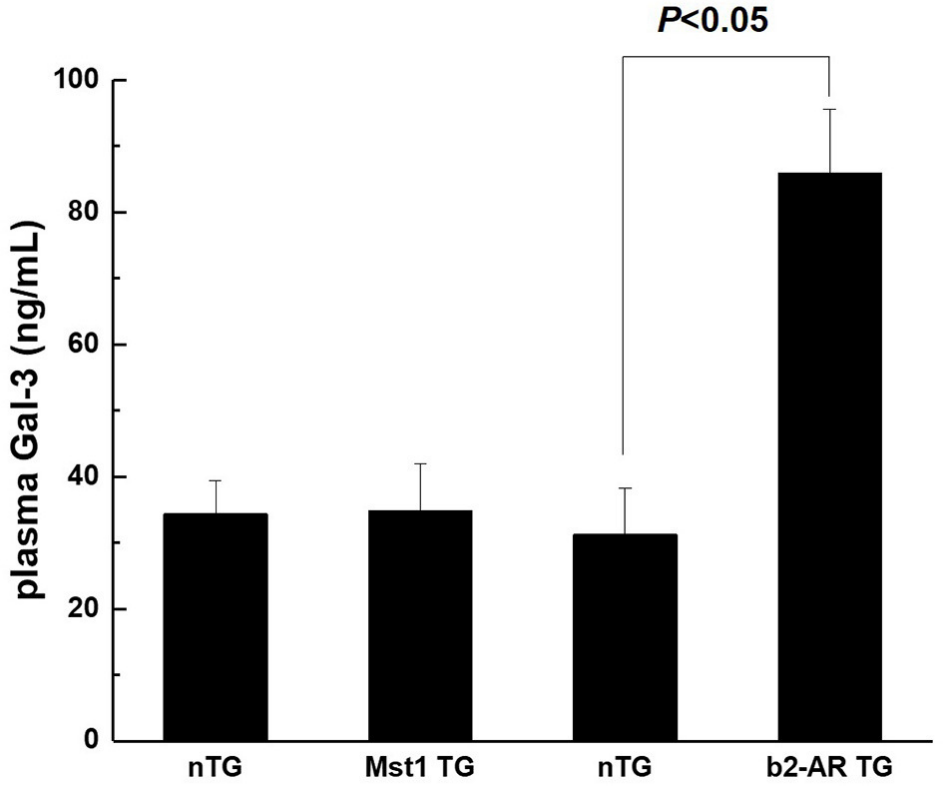
Plasma concentration of Gal-3 from nTG, Mst1-TG, and β2-AR-TG hearts in mice. Gal-3, galectin-3; nTG: non-transgenic mice; b2-AR TG, β2- adrenergic receptor transgenic; Mst1-TG, Mammalian sterile 20-like kinase 1 transgenic.

**Figure 2:**
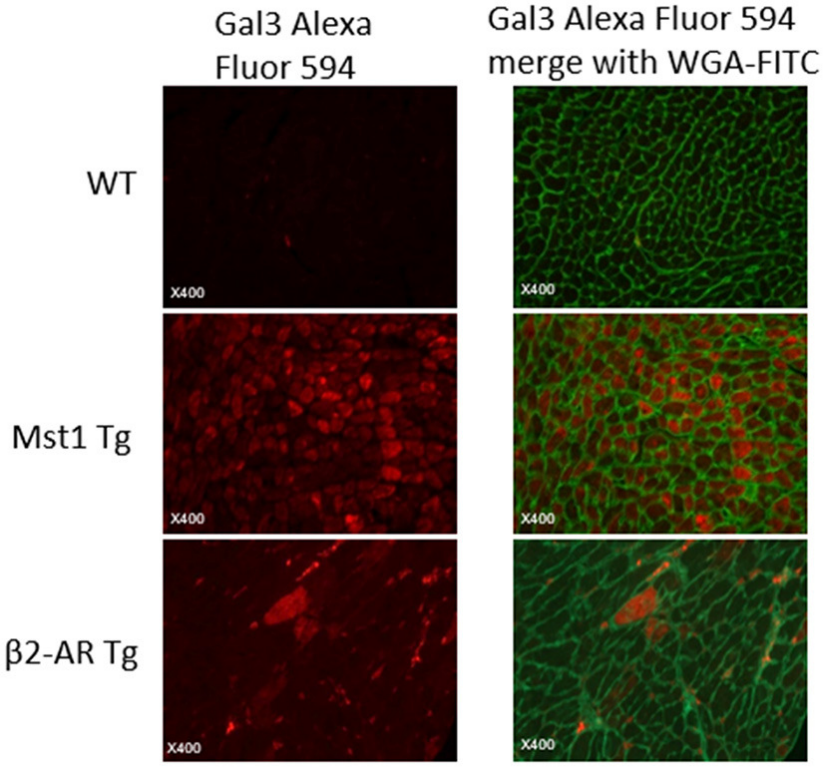
Immunofluorescent staining of LV sections from wild-type, Mst1-Tg, and β2-AR-Tg hearts in mice. Gal-3 staining: red fluorescence. Merged images also show cell boundaries in green (i.e., wheat-germ agglutinin-FITC staining).

Our previous studies showed that myocardial Gal-3 concentrations were higher in both Mst1-TG mice and β2-AR-TG mice compared to that in nTG mice (16, 19, 21). In keeping with our previous findings, the plasma Gal-3 concentration in β2-AR-TG mice was significantly elevated vs. nTG mice, whereas Mst1-TG mice showed no change in plasma Gal-3 concentration compared with that of respective nTG group (Figure 1). By immunohistochemistry, Gal-3 was localized in the cytoplasm and nucleus of cardiomyocytes, and often formed aggregates in Mst1-TG mice. Extracellular Gal-3 staining was uncommon within Mst1-TG mice. However, in β2-AR TG mice, although certain number of cardiomyocytes were positively stained by Gal-3, Gal-3 was more often expressed in interstitial cells (e.g., fibroblasts and macrophages) (Figure 2).

### Baseline Characteristics of HF Patients

The participants of this cohort study included 105 (63.2%) males and 61 (36.7%) females. Plasma Gal-3 concentrations were between 23.88–1157.63 pg/mL, and the median Gal-3 concentration was 158.42 pg/mL. All participants were divided into two groups according to the cut-off level of 158.42 pg/ml (below median Gal-3 and above median Gal-3). Next, the clinical data were compared between these two groups. As shown in Table 1, there were no statistical differences between two groups in terms of gender, hypertension history, DM history, treatments, and MACEs. However, patients with Gal-3 plasma concentrations above the median were older (*P* = 0.043). Table 1 also demonstrates that patients with increased plasma Gal-3 concentration had lower HB (*P* = 0.002) but higher plasma CR (*P* = 0.011), TIMP-1 (*P* = 0.002), LVESD (*P* = 0.036), LVESV (*P* = 0.043), and LVEDV (*P* = 0.036).

**Table 1:** Baseline characteristics of all HFrEF patients.

	**Below median Gal-3 (*n* = 83)**	**Above median Gal-3 (*n* = 83)**
Age (years)	60.6 ± 9.2	63.6 ± 9.3^*^
Gender (M/F)	55/28	50/33
Hypertension (%)	29.3	39.0
Diabetes mellitus (%)	7.2	7.2
Smoking (%)	57.8	47.0
Alcohol consumption (%)	41.5	41.0
Coronary heart disease (%)	34.9	48.2
HF history (years)	4.0 (2.0, 6.0)	4.0 (3.0, 7.0)
MLHFQ	25.0 (14.0, 34.0)	29.0 (16.0, 38)
SBP (mmHg)	122 ± 19	122 ± 23
DBP (mmHg)	77 ± 12	78 ± 11
CR (umol/L)	78.0 ± 12.1	84.3 ± 18.5^*^
BUN (mmol/L)	6.6 ± 1.5	7.1 ± 2.2
HB (g/L)	151.3 ± 21.7	141.9 ± 15.7^*^
AST (U/L)	24.6 ± 8.3	24.1 ± 9.4
ALT (U/L)	21.5 ± 10.6	20.9 ± 11.6
TIMP-1 (ng/mL)	113.3 ± 89.7	160.1 ± 103.7^*^
QT interval (ms)	419 ± 49	422 ± 50
BMI (Kg/m^2^)	22.9 ± 3.3	23.4 ± 3.9
Heart Rate (bpm)	78.5 ± 14.9	78.4 ± 18.3
LVEF (%)	35.3 ± 7.1	35.5 ± 7.7
LVESD (mm)	56.4 ± 8.6	57.3 ± 10.0^*^
LVEDD (mm)	68.9 ± 8.4	70.2 ± 9.4
LVESV (ml)	135 ± 55	156 ± 71^*^
LVEDV (ml)	199 ± 73	225 ± 85^*^
FS (%)	17.9 ± 4.1	17.8 ± 4.8
NYHA functional class		
I	20.5	10.8
II	53.0	60.2
III	22.9	26.5
IV	3.6	2.4
β-blocker (%)	79.5	81.9
ACEI/ARB (%)	90.4	89.2
MRA (%)	73.5	79.5
Digoxin (%)	19.3	27.7
Diuretics (%)	55.4	59.0
Death rate (%)	20.3	31.3
Re-hospitalization rate (%)	52.4	65.4
Composite-endpoint event (%)	61.4	72.3

*Data are mean (SD) or n (%) unless otherwise stated. F, female; M, male; MLHFQ, the Minnesota Living with Heart Failure Questionnaire; SBP, systolic blood pressure; DBP, diastolic blood pressure; CR, creatinine; BUN, urea nitrogen; HB, hemoglobin; AST, aspartate aminotransferase; ALT, alanine aminotransferase; TIMP-1, tissue inhibitor of metalloproteinases 1; BMI, body mass index; LVEF, left ventricular ejection fraction; LVESD, left ventricular end-systolic dimension; LVEDD, left ventricular end-diastolic dimension; LVESV, left ventricular end-systolic volumes; LVEDV, left ventricular end-diastolic volumes; FS, left ventricular fractional shortening; NYHA, New York Heart Association; ACEI, angiotensin converting enzyme inhibitor; ARB, angiotensin receptor blockers; MRA, mineralocorticoid receptors antagonist*;

* *P <0.05 vs. below median Gal-3 group*.

### Relationships Between Plasma Gal-3 Levels and Clinical Characteristics

**Figure 3:**
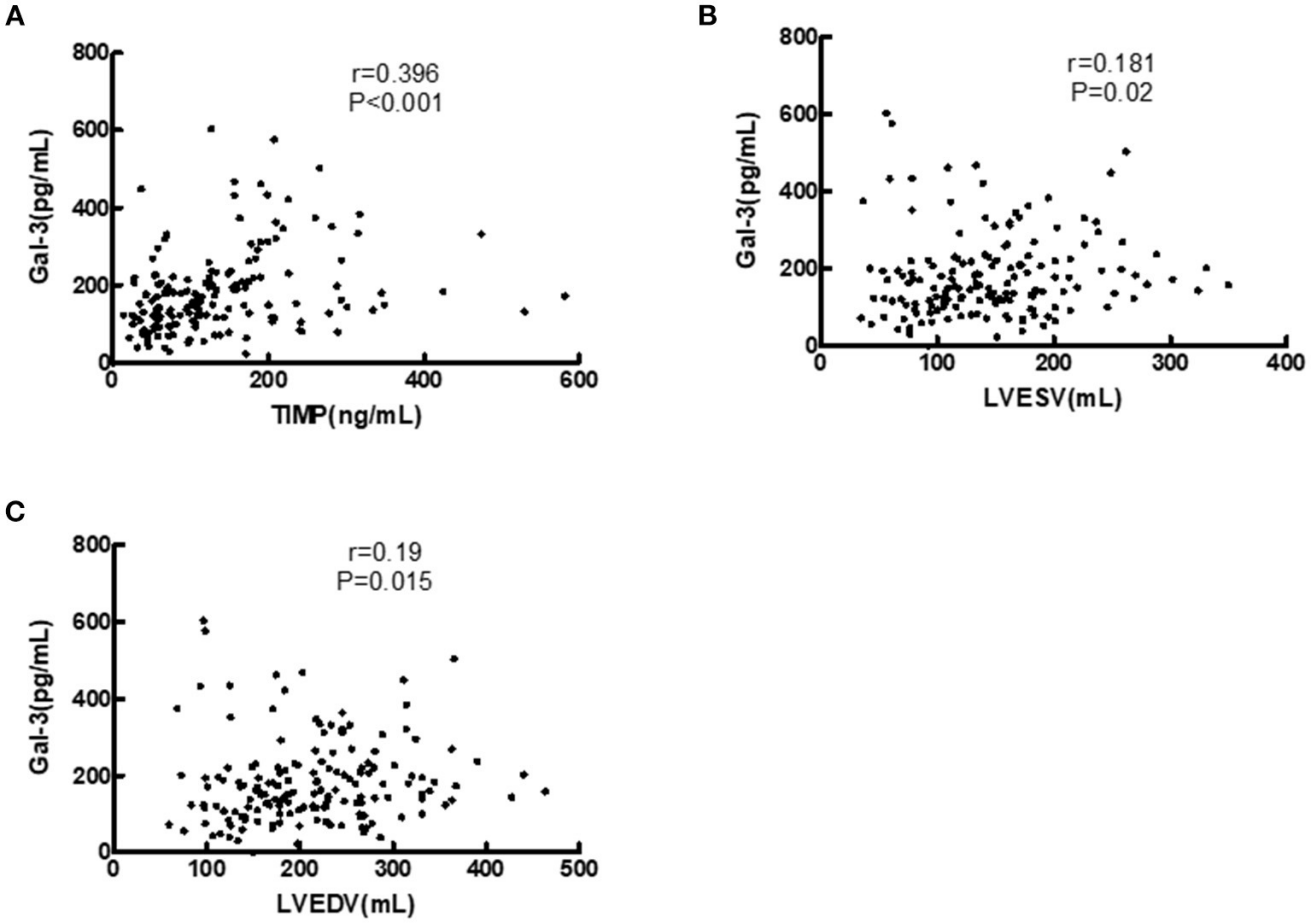
Correlations of serum Gal-3 levels with TIMP-1, LVEDV, and LVESV in HF patients. **(A)** Correlation of Gal-3 with TIMP-1. **(B)** Correlation of Gal-3 with LVESV. **(C)** Correlation of Gal-3 with LVEDV. Gal-3, galectin-3; LVESV, left ventricular end systolic volume; LVEDV, left ventricular end diastolic volume.

Figure 3 illustrates the associations between both Gal-3 and echocardiographic variables and the relationship between Gal-3 and TIMP-1. The results of spearman correlation analysis showed that, Gal-3 was positively correlated with TIMP-1 (*r* = 0.396, *P* < 0.001), LVESV (*r* = 0.181, *P* = 0.020), and LVEDV (*r* = 0.190, *P* = 0.015).

### Prognostic Value of Plasma Gal-3 Levels in all HFrEF Patients

**Figure 4:**
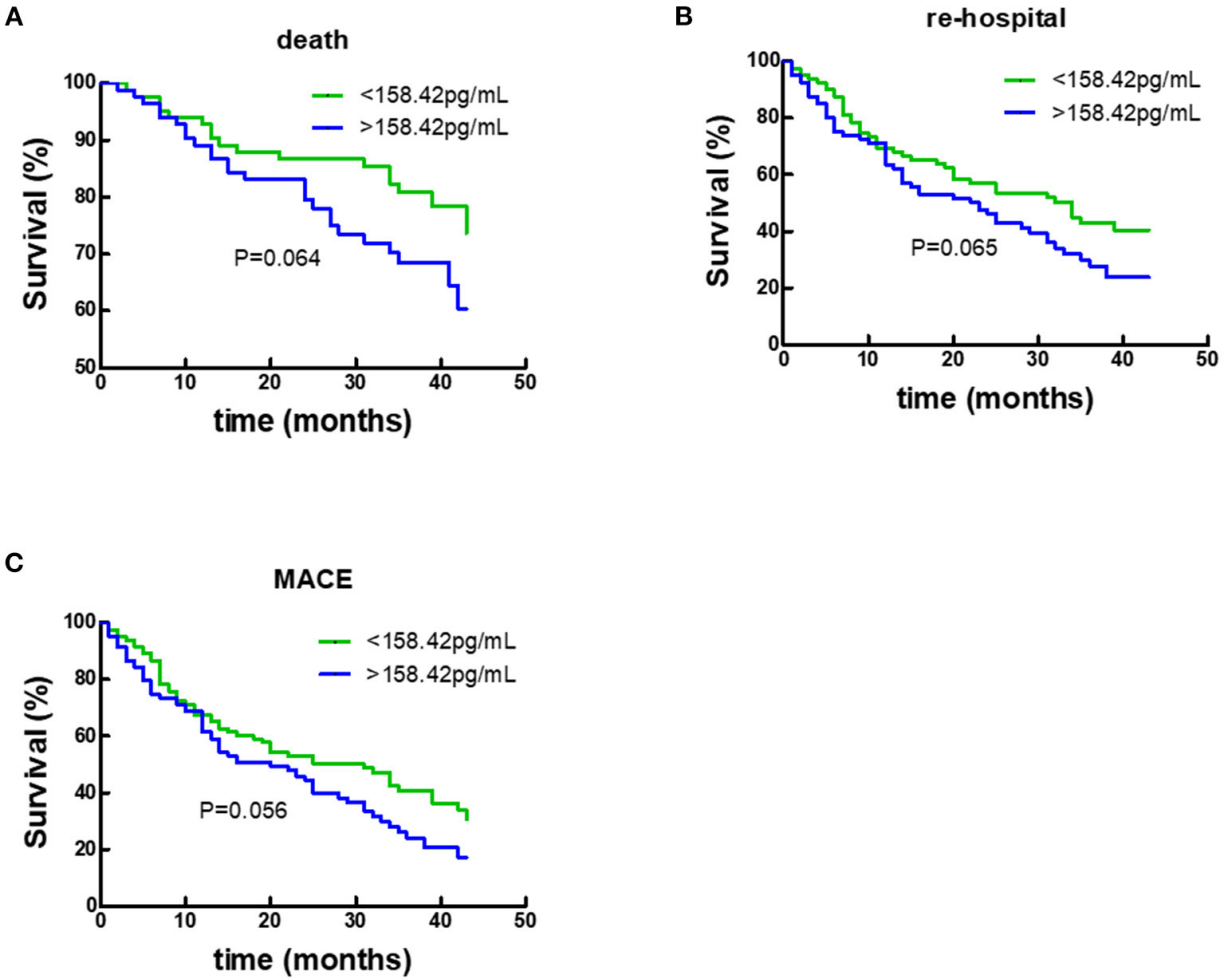
Kaplan–Meier survival curves according to baseline plasma Gal-3 levels in all HF subjects. **(A)** Death rates according to higher of lower baseline plasma Gal-3 levels. **(B)** Re-hospitalization rate according to higher of lower baseline plasma Gal-3 levels. **(C)** Composite-endpoint event rates according to higher of lower baseline plasma Gal-3 levels. Gal-3, Galectin-3.

During a 50-month follow-up, there were 43 deaths, 97 unplanned re-hospitalizations, and 111 composite endpoint events including death and unplanned re-hospitalizations. COX regression analysis and Kaplan-Meier analysis were performed. Following univariate Cox analysis, Gal-3 did not provide any prognostic value when all HF subjects were analyzed together (Figure 4; Table 2).

**Table 2:** Predictive value of baseline plasma Gal-3 to long-term outcomes in all HFrEF patients.

	**HR (95% CI)**	***P***
Death	1.769 (0.957, 3.268)	0.064
Re-hospitalization	1.454 (0.968, 2.184)	0.065
Composite-endpoint event	1.433 (0.983, 2.088)	0.056

*HR, hazard ratio; CI, confidence intervals*.

### The Comparison of Baseline Characteristics of HFrHF Patients With or Without CHD

Furthermore, we performed stratified analysis, respectively, in accordance with the two subgroups: the group with CHD and the other one without CHD. Gal-3 concentration was higher in subjects with CHD than without CHD (219.30 ± 20.73 pg/mL vs. 164.21 ± 8.85 pg/mL). Within HFrEF with CHD, there were no statistical differences between two groups in terms of age, gender, hypertension history, DM history, BUN, AST, ALT, QT intervals, LVEDD, LVESD, LVEDV, LVESV, treatments, and MACEs. However, Within HFrEF with CHD and Gal-3 plasma concentrations above the median patients had lower HB but higher plasma CR and TIMP-1. Whereas, within HFrEF patients with CHD, there were no statistical differences between two groups in terms of age, gender, hypertension history, DM history, BUN, AST, ALT, QT intervals, LVEDD, LVESD, and treatments. However, within HFrEF without CHD group, patients whose Gal-3 plasma concentrations above the median had lower HB but longer HF history, higher MLHFQ and death rate, larger LVEDV and LVESV (Table 3).

**Table 3:** Baseline characteristics of HFrEF with or without CHD patients.

	**HFrEF with CHD (*****n*** **=** **69)**		**HFrEF without CHD (*****n*** **=** **97)**	
	**Below median Gal-3**	**Above median Gal-3**	**Below median Gal-3**	**Above median Gal-3**
	**(*n* = 29)**	**(*n* = 40)**	**(*n* = 54)**	**(*n* = 43)**
Age (years)	63.8 ± 9.7	65.7 ± 9.3	58.9 ± 8.4	61.7 ± 9.1
Gender (M/F)	20/9	27/13	35/19	23/20
Hypertension (%)	50.0	38.5	18.5	18.6
Diabetes Mellitus (%)	10.3	12.5	5.6	2.3
Smoking (%)	62.1	52.5	55.6	41.9
Alcohol consumption (%)	37.9	40.0	43.4	41.9
HF history (years)	4 (2.8)	3 (2.7)	3 (1.25, 5)	5 (3, 7.75)^†^
MLHFQ	26 (10.41)	26 (11.37)	26 (16.34)	31 (23.39)^†^
SBP (mmHg)	126 ± 21	129 ± 22	120 ± 18	115 ± 22
DBP (mmHg)	78 ± 11	80 ± 10	77 ± 12	75 ± 11
CR (umol/L)	79.7 ± 14.0	88.8 ± 20.3^*^	77.1 ± 10.9	80.1 ± 15.7
BUN (mmol/L)	6.5 ± 1.4	7.2 ± 2.5	6.7 ± 1.5	7.1 ± 1.9
HB (g/L)	154.3 ± 21.6	144.3 ± 15.7^*^	149.6 ± 21.8	139.6 ± 15.7^†^
AST (U/L)	22.3 ± 7.6	24.5 ± 9.7	26.1 ± 8.6	23.3 ± 9.2
ALT (U/L)	20.2 ± 11.7	22.5 ± 12.6	22.3 ± 9.9	20.5 ± 9.8
TIMP-1 (ng/mL)	127.6 ± 115.9	183.3 ± 104.9^*^	105.6 ± 72.1	138.6 ± 99.0
QT interval (ms)	427 ± 48	407 ± 47	416 ± 48	429 ± 51
BMI (Kg/m2)	23.2 ± 3.6	24.3 ± 3.4	22.7 ± 3.2	22.5 ± 3.9
Heart Rate (bpm)	77.9 ± 13.2	78.6 ± 20.2	80.7 ± 25.2	76.2 ± 17.2
LVEF (%)	36.2 ± 7.3	38.2 ± 6.7	34.9 ± 7.0	33.0 ± 7.8
LVESD (mm)	55.0 ± 8.9	53.6 ± 9.3	57.2 ± 8.3	60.7 ± 9.6
LVEDD (mm)	67.5 ± 8.5	67.3 ± 8.5	69.7 ± 8.3	72.8 ± 9.6
LVESV (ml)	127 ± 57	137 ± 64	140 ± 54	172 ± 73^†^
LVEDV (ml)	190 ± 78	204 ± 93	204 ± 70.	244 ± 85^†^
FS (%)	18.0 ± 4.6	19.2 ± 5.1	17.7 ± 3.8	16.5 ± 4.1
NYHA functional class			
I	31.0	17.5	14.8	4.7
II	55.2	55.0	51.9	65.1
III	10.3	25.0	29.6	27.9
IV	3.4	2.5	3.7	2.3
β-blocker (%)	72.4	80.0	83.3	83.7
ACEI/ARB (%)	82.8	90.0	94.4	88.4
MRA (%)	72.4	82.5	74.1	73.7
Digoxin (%)	24.1	32.5	16.7	23.3
Diuretics (%)	48.3	52.5	59.3	65.1
Death rate (%)	20.7	22.5	20.4	39.5^†^
Re-hospitalization rate (%)	58.6	64.1	49.1	66.7
Composite-endpoint event (%)	65.5	70.0	59.3	74.4

*Data are mean (SD) or n (%) unless otherwise stated. F, female; M, male; MLHFQ, the Minnesota Living with Heart Failure Questionnaire; SBP, systolic blood pressure; DBP, diastolic blood pressure; CR, creatinine; BUN, urea nitrogen; HB, hemoglobin; AST, aspartate aminotransferase; ALT, alanine aminotransferase; TIMP-1, tissue inhibitor of metalloproteinases 1; BMI, body mass index; LVEF, left ventricular ejection fraction; LVESD, left ventricular end-systolic dimension; LVEDD, left ventricular end-diastolic dimension; LVESV, left ventricular end-systolic volumes; LVEDV, left ventricular end-diastolic volumes; FS, left ventricular fractional shortening; NYHA, New York Heart Association; ACEI, angiotensin converting enzyme inhibitor; ARB, angiotensin receptor blockers; MRA, mineralocorticoid receptors antagonist*;

* *P <0.05 vs. below median Gal-3 patients with CHD*;

† *P <0.05 vs. below median Gal-3 patients without CHD*.

### Prognostic Value of Plasma Gal-3 Levels in HFrHF Patients With or Without CHD

**Figure 5:**
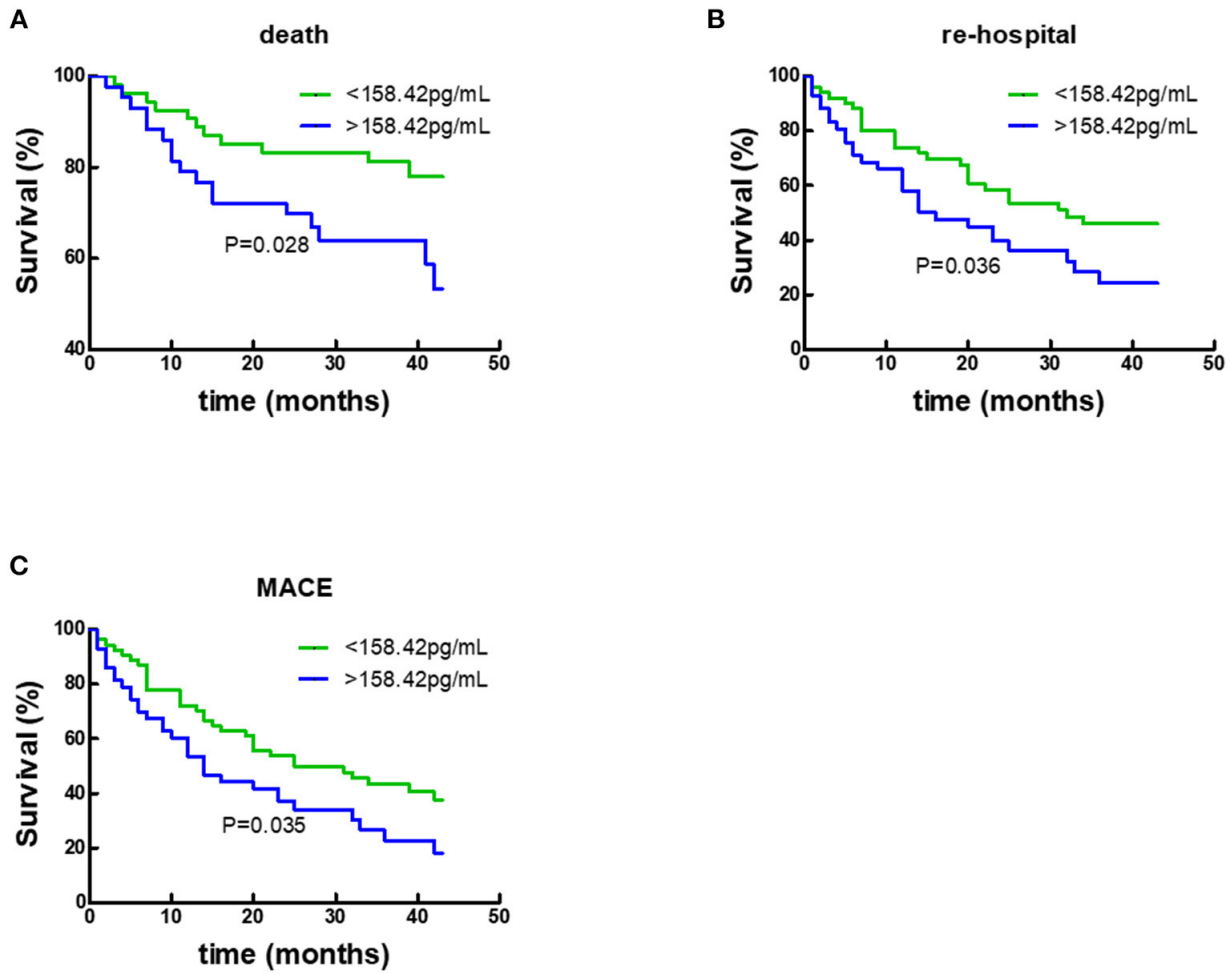
Kaplan–Meier survival curves according to baseline plasma Gal-3 levels in non-CHD subjects. **(A)** Death rates according to higher of lower baseline plasma Gal-3 levels. **(B)** Re-hospitalization rates according to higher of lower baseline plasma Gal-3 levels. **(C)** Composite-endpoint event rates according to higher of lower baseline plasma Gal-3 levels. Gal-3, Galectin-3.

By COX regression analysis and Kaplan-Meier analysis, the researchers of this study found that Gal-3 did not provide any prognostic value in participants with CHD. In contrast, as shown in Figure 5 and Table 4, respectively, Gal-3 did predict prognoses in HF subjects without CHD.

**Table 4:** Predictive value of baseline plasma Gal-3 to long-term outcomes in HFrEF with or without CHD patients.

	**HFrEF without CHD patients**		**HFrEF with CHD patients**	
	**HR (95% CI)**	***P***	**HR (95% CI)**	***P***
Death	2.292 (1.071, 4.905)	0.028	1.899 (0.664, 5.435)	0.232
Re-hospitalization	1.756 (1.021, 3.018)	0.036	1.473 (0.799, 2.716)	0.215
Composite-endpoint event	1.673 (1.022, 2.740)	0.035	1.545 (0.858, 2.780)	0.147

*HFrEF, heart failure with reduced ejection fraction; CHD, coronary heart disease; HR, hazard ratio; CI, confidence intervals*.

## Discussion

Our present study revealed four primary findings. First, Gal-3 levels in plasma were different between two mouse models of cardiomyopathy (i.e., Mst1-TG mice and β2-AR TG mice). Second, the expression of Gal-3 in myocardial tissue was different between these two mouse models. Third, plasma concentrations of Gal-3 were associated with TIMP-1 and echocardiographic parameters within HFrEF patients. Finally, although plasma concentrations of Gal-3 did not predict prognoses in all HFrEF participants, it had predictive power of prognoses in HFrEF without CHD subjects.

Gal-3 is primarily expressed in macrophages and fibroblasts, and is involved in myocardial fibrosis through activation of fibroblasts (6, 22). In the present study, we found that Gal-3 was also expressed in cardiomyocytes; moreover, the expression of Gal-3 was different between two mouse cardiopathy models. In Mst1-TG mice, Gal-3 was primarily expressed in cardiomyocytes, while it was mainly expressed in myocardial interstitial cells in β2-AR TG mice. This phenomenon has also been confirmed in our previous studies. We found that Gal-3 expression was confined to the infarcted area and was localized to both non-cardiomyocytes and cardiomyocytes in infartion-reperfusion (I/R) mice model, importantly, plasma levels of Gal-3 were also transiently elevated at three-days post-infarction. However, plasma Gal-3 was not elevated, despite of the increased Gal-3 mRNA and the protein levels in myocardial tissue within TAC mice (17). As we know, macrophages were regarded as the major cell type expressing Gal-3, in addition to cardiomyocytes and fibroblasts. The expression of Gal-3 in myocardial tissue was affected by inflammation (23, 24), β-adrenaline receptor pathway (25) and the Hippo pathway (22). We (18) found that in different cardiomyopathy models (Mst1-TG mice, β2-AR-TG mice, the I/R model and mice treated with isoproterenol), the density of macrophages within myocardial tissue was different between each other. Meanwhile, the increase of plasma Gal-3 was positively correlated with the increase of macrophages in these cardiomyopathy models. These results indicated that the expression of Gal-3 in myocardial tissue and plasma Gal-3 level depended on the etiology of HF. Although only two cardiomyopathy mice models were used in this study and there were not healthy participants as control to analysis the diagnostic value of Gal-3, to match with mice studies, these results could partially respond to the diagnostic value of plasma Gal-3 in HFrEF.

Myocardial fibrosis is an important pathophysiological mechanism involved in the development and progression of chronic heart failure (CHF) (26–28). Collagen synthesis by myocardial fibroblasts is activated in CHF and is affected by many determinants [e.g., Gal-3 (29, 30) and TIMP-1 (31–33)]. Some studies had disclosed that Gal-3 could stimulate myocardial fibrosis through various mechanisms (6, 10, 15, 30, 34) and Gal-3 deficiency ameliorates fibrosis and remodeling in dilated cardiomyopathy animal models (35–37). It has been demonstrated that TIMP-1 did contribute to ventricular remodeling and myocardial fibrosis in experimental HF models (33, 38). Since Gal-3 has been linked to myocardial fibrosis, it is plausible that elevated plasma concentrations of Gal-3 may also be linked to TIMP-1. In the present study, plasma Gal-3 was positively correlated with LVEDV and LVESV in chronic HFrEF patients. Few prior studies have systematically evaluated the relationship between echocardiographic measures and blood concentrations of Gal-3. The DEAL-HF trial performed serial echocardiographic measures in 240 HF patients with NYHA Class-III and -IV symptoms and found a positive association between increased plasma concentrations of Gal-3 and changes in LVEDV, whilst there was no correlation between baseline LVEDV and Gal-3 levels (39). These previous results are different from those of our present study. This discrepancy may be related to the different research subjects in each study. All subjects in the DEAL-HF trial were patients with NYHA Class-III and -IV symptoms, whereas all patients in our present study exhibited NYHA class I–IV symptoms. Therefore, future studies should further investigate the roles and mechanisms of Gal-3 in myocardial fibrosis and HF.

Although there have been many studies investigating the relationship between blood levels of Gal-3 and mortality in HF patients (40–42), the predictive value of Gal-3 for the prognosis of HF remains to be illusive (43). Recently, the PARADIGM-HF trial revealed that baseline and eight-month changes in serum Gal-3 levels did not predict outcomes in HFrEF patients (44). However, based on the results of animal experiments, it is speculated that the predictive value of Gal-3 in the prognosis of HF may be related to the etiology of HF (16–18) and the specific therapies used to treat HF (25). In the present study, we found that plasma Gal-3 did not predict the mortality in all HF subjects, while it did correlate with mortality in HF without CHD subjects. As some researchers proposed, due to different etiologies and underlying pathophysiological processes, HF was a heterogeneous disease. There were some results about the comparison of inflammation between CHD and non-CHD, and inflammation was less robust in non-ischaemic HF animal models than in ischaemic HF models. Plasma Gal-3 level may represent more serious damage of cardiomyocytes. To clarify the value of Gal-3 in HF, it may require preclinical investigations in more animal models of HF in addition to clinical studies.

### Limitations

We examined Gal-3 expression in mice and assessed the predictive value of blood Gal-3 on clinical endpoints in HFrEF patients. However, our present study had several limitations. First, only two cardiomyopathy mice models were used in this study, and we did not co-stain Gal-3 with macrophage or monocyte or fibroblast. Second, although 166 HFrEF patients were included, there were not healthy participants as control group to analysis the diagnosis value of Gal-3, which leads to a mismatch with mice studies. Third, the sample size that was small, needs to be enlarged in the future. Finally, we only assessed baseline Gal-3 concentrations in the present study, whereas broad assessment such as concentrations after treatments could be performed.

## Conclusions

Although plasma concentrations of Gal-3 were associated with TIMP-1 and echocardiographic parameters, the diagnostic and prognostic value of plasma Gal-3 in HFrEF were decided by the etiology of HF.

## Data Availability Statement

The raw data supporting the conclusions of this article will be made available by the authors, without undue reservation.

## Ethics Statement

The studies involving human participants were reviewed and approved by First Affiliated Hospital of Xi'an Jiaotong University. The patients/participants provided their written informed consent to participate in this study. The animal study was reviewed and approved by Xi'an Jiaotong University.

## Author Contributions

QL, X-JD, LB, and A-QM designed the study. QL, S-PC, YW, TL, Y-BX, JL, and XH followed up with the included patients. QL and Y-DS performed the experiments. QL and R-CZ collected and analyzed the data. QL prepared the manuscript. All authors read and approved the final manuscript.

## Funding

This study was supported by the Science and Technology Foundation of Shannxi Province (No: 2016HM-04 and S2017-ZDYF-ZDCXL-SF-0054) and the Clinical Research Award of the First Affiliated Hospital of Xi'an Jiaotong University, China (XJTU1AF2021CRF-016).

## Conflict of Interest

The authors declare that the research was conducted in the absence of any commercial or financial relationships that could be construed as a potential conflict of interest.

## Publisher's Note

All claims expressed in this article are solely those of the authors and do not necessarily represent those of their affiliated organizations, or those of the publisher, the editors and the reviewers. Any product that may be evaluated in this article, or claim that may be made by its manufacturer, is not guaranteed or endorsed by the publisher.

## Abbreviations

Gal-3: galectin 3
HF: heart failure
HFrEF: heart failure with reduced ejection fraction
ACEI: angiotensin converting enzyme inhibitors
ARB: angiotensin receptor blockers
CHD: coronary heart disease
MLHFQ: the Minnesota Living with Heart Failure Questionnaire
SBP: systolic blood pressure
DBP: diastolic blood pressure
CR: creatinine
BUN: urea nitrogen
HB: hemoglobin
AST: aspartate aminotransferase
ALT: alanine aminotransferase
TIMP-1: tissue inhibitor of metalloproteinases 1
BMI: Body mass index
LVEF: left ventricular ejection fraction
LVESD: left ventricular end-systolic dimension
LVEDD: left ventricular end-diastolic dimension
LVESV: left ventricular end-systolic volumes
LVEDV: left ventricular end-diastolic volumes
FS: left ventricular fractional shortening
NYHA: New York Heart Association
ARNI: Sacubitril/Valsartan
MRA: mineralocorticoid receptors antagonist
HR: hazard ratio
CI: confidence intervals
nTG: non-transgenic mice
β2-AR TG: β2- adrenergic receptor transgenic
Mst1-TG: Mammalian sterile 20-like kinase 1 transgenic.
